# Effects of COVID-19 on Vaccine-Preventable Disease Surveillance Systems in the World Health Organization African Region, 2020

**DOI:** 10.3201/eid2813.220088

**Published:** 2022-12

**Authors:** John Paul Bigouette, Anna W. Callaghan, Morgane Donadel, Angela Montesanti Porter, Louie Rosencrans, Jacquelyn S. Lickness, Sara Blough, Xi Li, Robert T. Perry, A.J. Williams, Heather M. Scobie, Benjamin A. Dahl, Jeffrey McFarland, Christopher S. Murrill

**Affiliations:** Centers for Disease Control and Prevention, Atlanta, Georgia, USA

**Keywords:** COVID-19, SARS-COV-2, polio, measles, rubella, surveillance, vaccine-preventable diseases, viruses, coronavirus disease, severe acute respiratory syndrome coronavirus 2

## Abstract

Global emergence of the COVID-19 pandemic in 2020 curtailed vaccine-preventable disease (VPD) surveillance activities, but little is known about which surveillance components were most affected. In May 2021, we surveyed 214 STOP (originally Stop Transmission of Polio) Program consultants to determine how VPD surveillance activities were affected by the COVID-19 pandemic throughout 2020, primarily in low- and middle-income countries, where program consultants are deployed. Our report highlights the responses from 154 (96%) of the 160 consultants deployed to the World Health Organization African Region, which comprises 75% (160/214) of all STOP Program consultants deployed globally in early 2021. Most survey respondents observed that VPD surveillance activities were somewhat or severely affected by the COVID-19 pandemic in 2020. Reprioritization of surveillance staff and changes in health-seeking behaviors were factors commonly perceived to decrease VPD surveillance activities. Our findings suggest the need for strategies to restore VPD surveillance to prepandemic levels.

Routine immunizations are among the most effective interventions to reduce illnesses, hospitalizations, and deaths from vaccine-preventable diseases (VPD) (*1*). The COVID-19 pandemic has directly affected services in health systems, including routine vaccine delivery, disease detection, laboratory confirmation of suspected cases, and outbreak responses (*2*–*4*). While the COVID-19 pandemic continues to disrupt routine immunization services, maintaining high-quality VPD surveillance is even more critical for detecting and responding effectively to VPD outbreaks, particularly in geographic areas with populations known to be vulnerable or low immunization coverage (*5*,*6*). 

Recent assessments have suggested that COVID-19 mitigation measures (e.g., social distancing, country border lockdowns, delays in specimen transportation) may have hindered detection and timely notification of VPD cases (*5*,*7*). It is unknown if the decrease in reported surveillance indicators and cases were because of interruptions in surveillance systems, decreases in disease occurrence, or both. This lack of data includes which specific activities of VPD surveillance were most disrupted by the COVID-19 pandemic at national and subnational levels and to what extent these disruptions varied by disease, location, or severity of impact. Information from frontline workers at the operational levels of VPD surveillance systems, such as STOP Program consultants, can serve as a foundational source to identify specific surveillance components affected by the COVID-19 pandemic. 

Since 1999, the STOP (originally Stop Transmission of Polio) Program has supported the Global Polio Eradication Initiative (GPEI) and national ministries of health to strengthen routine immunization and surveillance programs to reduce global VPD morbidity and mortality (*8*). STOP consultants are deployed through the World Health Organization (WHO) in 1 of 3 roles (communications specialist, data manager, or field epidemiologist) at national, provincial, district, and subdistrict levels (*8*). To help document the effect of the COVID-19 pandemic on general VPD surveillance, as well as on 3 priority surveillance systems—measles, rubella, and acute flaccid paralysis (AFP) for poliovirus—we solicited information from STOP consultants on disruptions they observed to VPD surveillance programs while deployed during 2020. We considered insights from STOP consultants relevant because of their field presence and direct involvement in immunization and VPD surveillance activities throughout the COVID-19 pandemic. 

## Methods 

We developed a web-based survey to determine how surveillance activities across different health system levels changed because of the COVID-19 pandemic. We distributed the survey in May 2021 to all 214 STOP consultants deployed to 44 countries across the 6 WHO regions who were on assignment from January 2020 through June 2021. Our report focuses on the WHO African Region, where 75% (160/214) of all STOP consultants were deployed. The survey questions primarily focused on their perceptions and observations about how COVID-19 had affected surveillance for poliovirus, measles, rubella, and other VPDs (pertussis, yellow fever, and neonatal tetanus) and asked STOP consultants to recall changes to their work in 2020 compared with 2019. The survey, developed in both English and French, included categorical responses and free-text fields and was sent to respondents by email with 2 weeks for completion.

We used R version 9.1.3 (The R Project for Statistical Computing, https://www.r-project.org) to conduct descriptive analyses to summarize the quantitative survey findings by calculating the proportion of responses to each question. We then reviewed qualitative information provided through the free-text responses to identify trends and themes that provided more context and descriptive information about the disruptions to VPD surveillance activities observed by the STOP consultants. 

## Results

Of the 160 STOP consultants in the African Region, 154 (96%) completed the survey (Table 1). Seventy percent (107/154) were epidemiologists, with 42% (64/154) stationed at the district level. All (154/154) African Region STOP consultants who responded were involved to some extent in COVID-19 activities in 2020, most often supporting response activities related to active surveillance and contact tracing; coordinating state-, district-, or local-level COVID-19 response efforts; engaging communities to implement preventive measures; and developing and disseminating COVID-19 weekly reports. 

**Table 1:** Characteristics of 154 STOP Program consultants in the World Health Organization’s African Region who responded to a survey on the effects of COVID-19 on vaccine-preventable disease surveillance systems, 2020*

Characteristics	Value†
Number of countries with STOP respondents	27 (57)
Median number of STOP respondents per country (IQR)	4 (2–8)
STOP consultant administrative level of assignment‡	
National	30 (19)
Provincial	53 (34)
District	64 (42)
Local	7 (5)
STOP consultant role	
Field epidemiologist	107 (70)
Communication specialists	26 (17)
Data manager	21 (14)

*Values are no. (%) except as indicated. IQR, interquartile range.

†Percentages may not equal 100% because of rounding.

‡Deployment location at time of survey response.

Among respondents, 97% observed that the COVID-19 pandemic either somewhat (54%; 83/154) or severely (43%; 66/154) affected measles or rubella surveillance in 2020. Themes from free-text responses suggested movement restrictions implemented to mitigate the risk of COVID-19 transmission affected the ability of surveillance staff to conduct supervisory visits, active case searches, suspected case investigations, community-based surveillance, and capacity-building activities and impeded collection and transportation of blood samples. About two thirds of the African Region STOP respondents (61%; 94/154) suggested that COVID-19 mitigation efforts also potentially disrupted the detection, notification, and reporting of measles or rubella cases.

Almost all of the African Region STOP consultants surveyed observed that AFP surveillance for poliovirus was either somewhat (55%; 84/154) or severely (43%; 66/154) affected by the COVID-19 pandemic during 2020 (Table 2). Some respondents suggested that country lockdowns and the reassignment of polio program staff to the COVID-19 response contributed to the decrease in AFP surveillance activities in 2020. Surveillance activities delayed by COVID-19 mitigation measures commonly mentioned by respondents included active case investigations and transporting samples to national laboratories for processing. 

**Table 2:** Impact of COVID-19 on measles and rubella, AFP/polio, and other VPD surveillance systems according to 154 STOP Program consultants in the World Health Organization’s African Region, 2020*

Category	No. (%) respondents		
	Measles and rubella	AFP/polio	Other VPDs
Compared to 2019, in 2020 did COVID-19 impact surveillance overall?			
Severely impacted	66 (43)	66 (43)	38 (25)
Somewhat impacted	83 (54)	84 (55)	92 (60)
Not at all impacted	3 (2)	3 (2)	15 (10)
Does not know	2 (1)	1 (1)	9 (6)
Missing response	0	0	0
Compared to 2019, in 2020 did you conduct active surveillance?			
Increased active surveillance	3 (2)	1 (1)	2 (1)
Same level of active surveillance	21 (14)	19 (12)	24 (16)
Decreased active surveillance	108 (70)	119 (77)	100 (65)
No active surveillance	20 (13)	13 (8)	24 (16)
Does not know	0	0	0
Missing response	2 (1)	2 (1)	4 (3)
Compared to 2019, in 2020 did staffing change for surveillance at your level of responsibility?			
Staffing increased	4 (2)	11 (7)	4 (3)
No change in staffing	103 (67)	89 (58)	107 (69)
Staffing decreased	23 (15)	31 (20)	24 (16)
Does not know	23 (15)	22 (14)	18 (12)
Missing response	1 (1)	1 (1)	1 (1)
Compared to 2019, in 2020 did COVID-19 impact adversely the detection, notification and reporting of cases?			
Yes	94 (61)	106 (69)	94 (61)
No	37 (24)	40 (26)	39 (25)
Does not know	22 (14)	7 (5)	20 (13)
Missing response	1 (1)	1 (1)	1 (1)
For responses indicating an adverse impact on the detection, notification, and reporting of VPD cases, identify the health care level impacted (select all that apply)			
At the national level	24 (26)	37 (35)	29 (30)
At the province level	40 (43)	50 (47)	45 (48)
At the district level	75 (80)	81 (76)	72 (77)
At the local level	86 (92)	92 (87)	85 (90)
Compared to 2019, in 2020 did COVID-19 mitigation efforts disrupt the transport of specimens of suspected VPD cases to relevant who reference laboratories?			
Yes	50 (32)	73 (47)	42 (27)
No	50 (32)	36 (23)	44 (29)
Does not know	52 (34)	44 (29)	66 (43)
Missing response	2 (1)	1 (1)	2 (1)

*AFP, acute flaccid paralysis, VPD, vaccine-preventable disease.

†Percentages may not equal 100% because of rounding.

African Region STOP consultants noted the number of active AFP surveillance activities they conducted decreased (77%; 119/154) or were not completed (8%; 13/154) in 2020 compared with 2019. Respondents also noted that lockdowns to mitigate the transmission of COVID-19 often reduced the public’s access to and use of local healthcare facilities and services, which may have allowed AFP cases to go undetected. 

Most respondents felt that COVID-19 either somewhat (60%; 92/154) or severely (25%; 38/154) affected surveillance of other VPDs, such as pertussis, yellow fever, and neonatal tetanus, in 2020. Specifically, 65% (100/154) of respondents suggested that active surveillance conducted for other VPDs decreased in 2020 compared with 2019; 16% (24/154) noted they did not conduct any active surveillance for these other VPDs in 2020. However, African Region STOP consultants did observe some improvements in measles or rubella surveillance in 2020, including the integration of VPD surveillance activities into COVID-19 response activities; using phone, WhatsApp, and similar technologies for communication when field access was limited; improving community-based surveillance; and implementing VPD surveillance refresher trainings for health facility staff. 

## Discussion

As the number of undervaccinated children increases because of disruptions in routine immunization services, understanding how VPD surveillance systems have fared during the COVID-19 pandemic becomes critical (*1*). Descriptions collected from WHO African Region STOP consultants support general observations that the COVID-19 pandemic adversely affected implementation of VPD surveillance activities in 2020 and that COVID-19 mitigation strategies and staff reassignments may have been substantial disrupting factors. Further efforts are needed to directly associate observations from our survey with changing trends in indicators of disease surveillance systems in the WHO African Region and across other WHO regions during 2020. 

STOP consultants spend most of their time at local or district levels, and results from this survey suggest that certain VPD surveillance activities such as detection, notification, and reporting were affected primarily at those levels; however, some effect was observed on regional and national activities in many WHO African Region countries because the COVID-19 response was prioritized. Respondents also noted that measles and rubella surveillance staff were often shifted to COVID-19–related activities. In countries where staffing and funding are limited, integrating VPD surveillance functions across systems, instead of relying on standalone systems, could be a mechanism to address resource limitations, even moreso as COVID-19 becomes endemic in countries.

In addition, some respondents indicated that country lockdowns and movement restrictions contributed to delays in suspected case investigations and transportation of samples to reference laboratories. Although the Global Polio Eradication Initiative had published interim guidance on how to continue conducting polio surveillance with COVID-19 mitigation measures in place (*9*,*10*), survey responses seem to corroborate existing reports of decreased AFP surveillance indicators during the COVID-19 pandemic (*7*). Although AFP surveillance for poliovirus may have been adversely affected in many countries by the pandemic, it is notable that AFP surveillance systems were still able to identify circulating vaccine-derived and wild poliovirus in the African Region (*11*). 

Among limitations in our survey, consultant-respondents were asked to respond to questions based on their personal knowledge and experience, which is likely to have varied depending on location, length of time in assignment, and type of assignment, and should be considered a collection of observations or case series. These responses provide a valuable starting point to generate hypotheses for future public health investigations and research into impacts of VPD surveillance systems during the COVID-19 pandemic. Second, each STOP consultant surveyed was contracted through WHO to work in partnership with the Ministry of Health in their country of assignment. Thus, responses might not represent observations of the COVID-19 pandemic’s effect on VPD surveillance activities among private sector or non–Ministry of Health immunization and surveillance staff, or in countries without STOP consultants. 

## Conclusions

Since 2020, the COVID-19 pandemic has disrupted or delayed many aspects of both aggregate and case-based VPD surveillance systems in Africa. A conscientious effort should be made to connect COVID-19 response activities to existing public health surveillance systems, especially where substantial investments have been made towards strengthening VPD contact tracing, active surveillance, and specimen collection and transportation. Efforts to integrate systems across diseases would promote and facilitate efficient restructuring, integration, and coordination of surveillance systems globally. 
